# Investigation of angiotensin-1 converting enzyme 2 gene (G8790A) polymorphism in patients of type 2 diabetes mellitus with diabetic nephropathy in Pakistani population

**DOI:** 10.1371/journal.pone.0264038

**Published:** 2022-02-17

**Authors:** Hooria Younas, Tahira Ijaz, Nakhshab Choudhry

**Affiliations:** 1 Department of Biochemistry, Kinnaird College for Women, Lahore, Punjab, Pakistan; 2 Department of Biochemistry, King Edward Medical University, Lahore, Punjab, Pakistan; Indiana University Purdue University at Indianapolis, UNITED STATES

## Abstract

**Background:**

Type 2 diabetes mellitus is a multifactorial disease that escalates the risk of other associated complications such as diabetic neuropathy, retinopathy, and nephropathy. Diabetic nephropathy is a microvascular condition that leads to end-stage renal disease (ESRD). There are several genes involved in disease development and it is a challenging task to investigate all of these. Nonetheless, identifying individual gene roles can assist in evaluating the combinatorial effects with other genes. Angiotensin-1 converting enzyme 2 (ACE2), is the key regulator of blood pressure in the Renin-Angiotensin-Aldosterone System that hydrolyzes angiotensin II (vasoconstrictor) into angiotensin 1–7 (vasodilator). The association of different variants of the *ACE2* with the risk of type 2 diabetes mellitus has been determined in various populations with susceptibility to other complications. This study was aimed to investigate the association of Angiotensin-1 converting enzyme 2 polymorphism, G8790A, with the increased risk of type 2 diabetes mellitus (T2DM) development with the complication of diabetic nephropathy (DN) in the Pakistani population.

**Methods:**

In this case-control study, a total of 100 healthy controls and 100 patients of type 2 diabetes mellitus aged > 40 years, having disease duration ≥ 10 years were compared. The G8790A polymorphism in *ACE2* was analyzed by allele-specific polymerase chain reaction (AS-PCR). The urinary albumin excretion (UAE), urinary creatinine, and albumin to creatinine ratios (ACR) were determined to assess renal function status. Pearson bivariate correlation coefficients were calculated to investigate the relationship among all the parameters. Crude and adjusted odds ratios were found to determine any risk association between *ACE2* G8790A polymorphisms and disease development. The p-values < 0.05 were considered significant.

**Results:**

A homogeneity was obtained regarding the distribution of data by sex, BMI, diastolic blood pressure, pulse rate and urinary creatinine levels between case and control groups. The ACR showed a significant correlation with UAE (r = 0.524, p = 0.001), urinary creatinine (r = -0.375, p = 0.001) and random blood sugar levels (r = 0.323, p = 0.005) with the complication of diabetic nephropathy in T2DM patient. Females with the AA genotype had a 10-fold increased risk for the development of type 2 Diabetes (OR = 9.5 [95% CI = 2.00–21.63] p<0.002). Males having A allele showed a significant association for susceptibility of type 2 Diabetes (OR = 3.807 [95% CI = 1.657–8.747] p<0.002). However, none of the genotypes or alleles revealed an association for diabetic nephropathy in male and female patients. Urinary ACR was also found to be positively correlated with UAE (r = 0.642 p = 0.001 & 0.524, p = 0.001) and random blood sugar levels (r = 0.302, p = 0.002 & r = 0.323, p = 0.005) in T2DM and T2DM+DN groups, respectively.

**Conclusion:**

The study finding indicated that female AG/AA genotype and male A genotype of G8790A polymorphism in the *ACE2* gene were associated with type 2 diabetes mellitus as a genetic risk factor but are not associated with diabetic nephropathy in the Pakistani population.

## Introduction

Diabetes Mellitus is considered a major cause of morbidity and mortality in both developed and developing countries. Diabetes may lead to many vascular complications like hypertension, neuropathy, nephropathy, retinopathy and ischemic heart disease. Diabetic nephropathy is the most common underlying cause of chronic kidney disease (CKD) and its subsequent progression to end-stage renal disease (ESRD) requiring renal replacement therapy (RRT) in the form of dialysis or transplant [1]. Baseline albumin excretion, smoking, hyperglycemia, high blood pressure and serum lipid profile are considered as modifiable risk factors while advanced age, diabetes duration, race, ethnicity and genetic profile are non-modifiable risk factors of nephropathy [2]. The implication of renin-angiotensin-aldosterone system (RAAS) components in the pathophysiology of diabetes-induced nephropathy have been well known for many years.

RAAS is involved in diabetic nephropathy by triggering glomerular efferent arteriolar constriction and intraglomerular pressure. The angiotensin I is converted into angiotensin II by angiotensin-converting enzyme (ACE) in RAAS. Angiotensin II (Ang II), a key component of RAAS, accelerates kidney damage directly by promoting inflammation, fibrosis, proliferation and hypertrophy of renal parenchyma. Angiotensin II as vasoconstrictor and stimulator of aldosterone synthesis, increases glomerular hydrostatic pressure and ultrafiltration of plasma proteins mainly through post-glomerular arteriolar constriction and thus lead to onset and development of nephropathy [3]. The contribution of genetic polymorphisms of the entire intra-renal renin-angiotensin-aldosterone system in the pathogenesis of diabetes and diabetic nephropathy is provided by extensive clinical and experimental data [4, 5]. In recent years, numerous studies have highlighted the fact that specific RAAS genes variants may confer an increased risk for diabetic complications such as coronary heart disease, hypertension, cerebral stroke, retinopathy, and nephropathy [6].

One of the most widely studied polymorphism within the RAAS is G8790A polymorphism, a single nucleotide polymorphism (SNP) in the third intron of the Angiotensin-I converting enzyme 2 gene (*ACE2*) that encodes for Angiotensin -1 converting enzyme 2 (ACE2). The *ACE2* gene is located on chromosome Xp22.2 and consists of 18 exons which span about 41kb of genomic DNA. It was first discovered in 2000 as a key regulator of blood pressure by influencing the heart and renal functions. ACE2 regulates AngII levels within the renal glomerulus by converting AngII into Ang 1–7 in proximal renal tubular cells. Ang 1–7 has shown a renoprotective effect by acting as vasodilator, anti-proliferator, an inhibitor of fibrosis and O_2_ consumption, stimulator of prostaglandin E (PGE), diuretic and natriuretic, and elevating secretion of prostacyclin and NO. Thus, its function is to counterbalance the effect of ACE [7]. In pancreatic islets, regulation of Ang II and/or Ang-(1–7) levels by ACE and ACE2 controls insulin secretion to such extent that blood flow is influenced by local levels of angiotensin peptides [8]. A depletion of the ACE2 enzyme in pancreatic tissue might contribute to less insulin secretion, leading to diabetes mellitus. In the renal glomerulus, ACE2 deficiency caused impaired degradation of Angiotensin II which gets accumulated in the glomerular part and locally accelerates proteinuria leading to glomerulosclerosis [9]. The action of ACE and ACE2 in the kidneys may determine a decline in glomerular filtration rate at advanced stages of diabetes mellitus [10]. Improvement in fasting blood glucose levels, glucose tolerance, high β-cell proliferation accompanied along reduced β-cell apoptosis and elevated first-phase insulin secretion was seen in the db/db mice with ACE2 gene therapy [11]. ACE2/Ang-(1–7)/MAS axis serves as a negative regulator for the RAAS. These protective effects give a rationale for implementing strategies that might enhance ACE2 in diabetics by gene therapy [12].

The G8790A polymorphism of *ACE2* has been emphasized in several studies where at 8790^th^ position, base guanine (G) is substituted by adenine (A) in the third intron. This substitution altered mRNA pattern and *ACE2* gene expression. *ACE2* G8790A SNP has been shown to be associated with hypertension, cerebral cronary and coronary heart disease in T2DM patients [8, 13]. Many studies yielded contradictory findings concerning the role of *ACE2* G8790A polymorphism in T2DM associated complications in different populations and ethnic groups. G8790A polymorphism association with diabetes complications had been studied in populations of Finland [5], South Asians Odisha, India [14], Caucasians [15] and British Isles [16]. Although it is not sure whether this polymorphism also influences susceptibility to diabetic nephropathy in type 2 diabetic patients or not. The purpose of this case-control study was to elucidate the association of *ACE2* G8790A polymorphism with susceptibility to increased risk of diabetic nephropathy as a complication of type 2 diabetes mellitus in the Pakistani population.

## Materials and methods

### Ethics statement

The Institutional Review Board of Kinnaird College for Women, Lahore approved the conduct of this case-control analytical study. All the Ethical Principles of the World Medical Association Declaration of Helsinki for Medical Research which involves Human Subjects, were followed. Written informed consent forms were obtained from all the participants after a brief explanation of the research work.

### Study subjects

In this study, 200 individuals including 100 with type 2 diabetes mellitus (T2DM) and 100 healthy individuals with no previous history of diabetes or any other disease were enrolled (S1 Table). Diagnosed patients of T2DM (aged 40) having more than 10 years of disease history including both sexes (equal numbers) were enrolled from the Diabetes Management Centre (DMC) of Services Hospital Lahore. Patients with hematuria, pyuria and end-stage renal disease (ESRD) were excluded. Age and sex-matched healthy subjects without a history of diabetes mellitus and any other illnesses were selected as controls from the general population. For each participant, demographic and clinical data were obtained with anthropometric (weight, height and body mass index) and vital sign (Blood pressure, pulse rate and temperature) measurements. Blood (3 ml) was drawn by venipuncture and collected in EDTA vacutainer. Urine samples were collected in urine containers and centrifuged at 4000 rpm for 5 minutes in Falcon tubes. The supernatant was stored in labelled Eppendorf tubes at -70°C till further analysis of proteinuria (creatinine and albumin). Creatinine and albumin were estimated using commercially available kits (Human, Germany) on HUMA–STAR 600 automated clinical chemistry analyzer in each urine sample. ACR (Albumin to creatinine ratio) was calculated to determine renal status.

### Genotype analysis of the *ACE2* G8790A polymorphism

Genomic DNA was extracted by standard phenol/chloroform method from a blood sample [17] and stored at -70°C and polymorphism was determined by AS-PCR [18] in the Biochemistry Lab of Kinnaird College Women Lahore. The PCR primers were 5’-TGCTTATTACTTGAACCAGGGA**A-**3’ (*ACE2*_AF), 5’-ATGCTTATTACTTGAACCAGGGA**G**-3’ (*ACE2*_GF) and 5’-GCCCAGAGCCTCTCATTGTA-3’ (*ACE2*_Out-R). The Batch Primer3 software was used to design Allele-specific primers for *ACE2* G8790A polymorphism [19]. By UCSC Genome Browser, *In-silico* PCR was performed to find out the product size and accuracy of primers (https://genome.ucsc.edu/cgi-bin/hgPcr) [20]. Oligocalc software was utilized to determine all parameters (GC%, Tm, length, self-complementary) of these primers (http://biotools.nubic.northwestern.edu/OligoCalc2.0.html) [21]. Two PCR reactions for each sample were performed, one having forward primer for A- allele (*ACE2*-AF) with common reverse primer (*ACE2*_out_R) and the other having forward primer for G-allele (*ACE2*-GF) with common reverse primer (*ACE2*_OUT_R). PCR reaction mixture was consisted of 2μl of genomic DNA template, 2.5μl of 25mM MgCl_2_, 10pmol of forward and reverse primers (1μl), 10μl of Master Mix (MERK DYE), and 3.5μl of autoclaved distilled H_2_O in the total volume of 21μl. PCR was performed on thermocycler (Bio-Rad, USA) with following conditions: initial denaturation step (Temp: 95°C; Time: 1m) followed by 35 cycles of denaturation (Temp: 95°C; Time: 45s, annealing (Temp: 63°C; Time: 45s), extension (Temp: 72°C; Time: 1m), and a final extension step (Temp: 72°C; Time: 5m). The PCR products were analyzed on 1.5% Agarose Gel Electrophoresis.

### Statistical analysis

The statistical analyses were carried out using SPSS, version 21 for windows (SPSS, Chicago, IL, USA). Continuous variables data were presented as numbers, percentages and mean ± standard deviation (SD). These variables were analyzed in comparison between T2DM patients by student t-test in males and by one way ANOVA in females. The p-value < 0.05 was considered significant. Categorical variables (genotypes and alleles frequencies) were explored by Chi-square (χ^2^) test. All the clinical parameters were correlated with ACR through Pearson correlation coefficient. Odds ratios (ORs) and 95% confidence intervals (CIs) were also calculated to estimate the strength of association where non-risk allele or genotype was considered as reference. The multinomial logistic regression was applied to adjust the odds ratios (OR^A^) by considering age, BMI, Smoking status (smokers/Non-smokers) and albuminuric (normoalbuminuric; ACR < 30 mg/g, microalbuminuric; ACR 30–299 mg/g and macroalbuminuric; ACR > 300 mg/g) as confounding factors. As the *ACE2* locus is on the X–chromosome, analysis of data for each sex was performed separately.

## Results

### Demographic, clinical and biochemical characteristics of the study population

The demographic, clinical and biochemical parameters of studied groups (controls and type 2 diabetes mellitus patients; T2DM) were compared and data is shown in Table 1. Out of 100 T2DM patients, 75 (75%) were micro/macroalbuminuric and thus considered as type 2 diabetes mellitus patients with diabetic nephropathy (T2DM+DN) while 25 (25%) were normoalbuminuric which were labelled as type 2 diabetes mellitus without diabetic nephropathy (T2DM-DN). There were no significant (p-value > 0.05) differences observed in the Body mass index (BMI), Diastolic blood pressure (DBP), pulse rate (PR) and urinary creatinine (UC) between controls and patients. Moreover, there were no significant differences present in the duration of T2DM, BMI, SBP, DBP, urinary creatinine between T2DM patients with or without diabetic nephropathy (p-value>0.05). The results for the above-mentioned variables indicated homogeneity of distribution between T2DM and control groups likewise for T2DM+DN and T2DM-DN groups. However, age, smoking status, systolic blood pressure (SBP), random blood sugar (RBS), urinary albumin excretion (UAE), and albumin to creatinine ratio (ACR) (p-value<0.002) showed highly significant differences in data between controls and cases.

**Table 1:** Demographic features, anthropometric measurements and clinical parameters of controls and patients involved in the present study.

Characteristics:	Control (N = 100)	T2DM (N = 100)	p-value	T2DM (-DN) (N = 25)	T2DM (+DN) (N = 75)	p-value
**Sex**						
**Male**	50 (100%)	50 (100%)	1.000	14 (28%)	36 (72%)	0.488
**Female**	50 (100%)	50 (100%)		11 (22%)	39 (78%)	
**Age (years)**	50.22±6.40	55.28±7.39	<0.001**	57.28±7.32	54.61±7.35	0.119
**BMI (Kg/m** ^**2**^ **)**	27.99±3.44	28.71±6.28	0.314	28.00±5.64	28.94±6.50	0.550
**Smoking Status**						
**Smokers**	9 (9%)	0 (0%)	0.002**	3 (33%)	6 (67%)	0.545
**Non-Smokers**	91 (91%)	100 (100%)		22 (24%)	69 (76%)	
**T2DM duration (<10) years)**	-----	14.6±4.74	<0.001**	14.08±4.15	14.77±4.94	0.529
**SBP (mmHg)**	120.00±0.00	123.66±14.28	0.010**	118.80±14.24	125.28±14.01	0.049*
**DBP (mmHg)**	80.00±0.00	79.10±8.539	0.293	76.40±9.95	80.00±7.88	0.068
**Pulse Rate (per minute)**	79.210±6.36	78.7500±6.379	0.610	75.560±6.16495	79.813±6.12636	0.003**
**Random Blood Sugar (mg/dl)**	115.030±14.79	237.4800±91.275	0.001**	224.240±71.07172	241.893±97.10103	0.405
**Urinary creatinine (mg/dl)**	90.43±44.14	98.26±61.18	0.301	107.67±61.71	95.13±61.10	0.377
**UAE (mg/l)**	11.35±39.61	141.98±143.12	<0.001**	12.43±9.40	185.17±140.77	<0.001**
**ACR (mg/g)**	26.10±159.21	186.31±212.89	<0.001**	13.30±7.16	243.98±217.10	<0.001**

Data is given as Mean ± SD. BMI; body mass index, SBP; systolic blood pressure, DBP; diastolic blood pressure, UAE; urinary albumin excretion, ACR; Albumin to creatinine ratio, T2DM; type 2 diabetes mellitus, T2DM-DN; type 2 diabetes mellitus without nephropathy, T2DM+DN; type 2 diabetes mellitus with nephropathy.

*significant at the level of 0.05 and

**highly significant at the level of 0.01.

As expected, systolic blood pressure (SBP) and clinical data for albumin (UAE) and albumin-creatinine ratio (ACR) were found to have statistically significant variations in T2DM groups (T2DM+DN and T2DM-DN) (p-value < 0.05). A highly significant difference of data distribution in age (males: 57.84±7.34 & females: 52.88±7.15) and smoking status (males: 9% & females: 0%) was observed between males and females (p-value<0.01) of the case group while the data for all other variables was consistently distributed by gender in both case and control groups (S2 Table).

### Correlation of ACR with age, BMI and other clinical parameters

**Fig 1:**
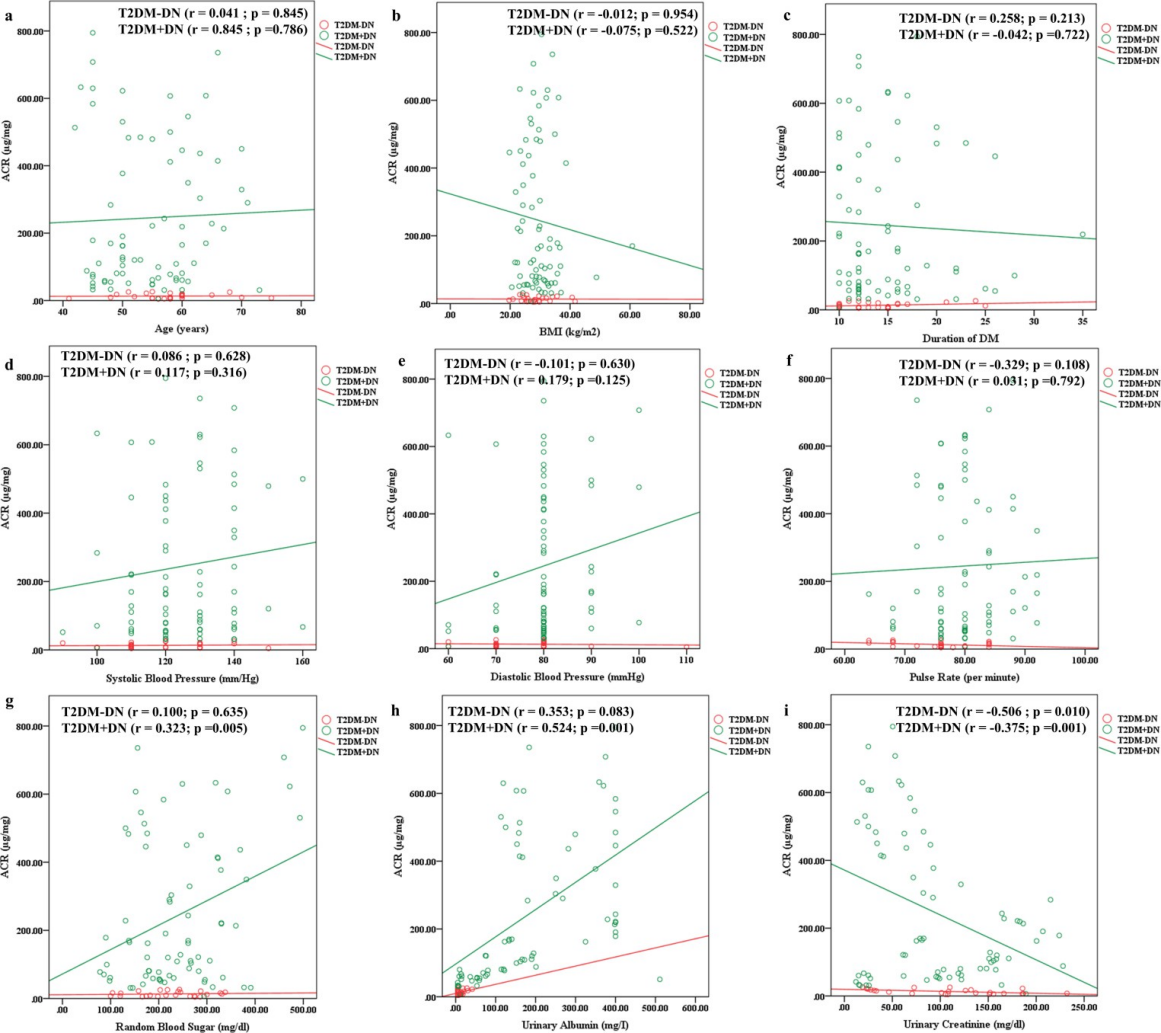
Scatter plots showing Pearson correlation coefficients of ACR with various clinical parameters among type 2 diabetes mellitus patients with or without diabetic nephropathy. Correlation of ACR with a) age b) BMI c) Duration of Diabetes mellitus d) Systolic blood pressure e) Diastolic blood pressure f) pulse rate g) random blood sugar h) urinary albumin excretion i) urinary creatinine.

We had analyzed the correlation coefficients of ACR with age, BMI and clinical parameters among the participants with or without type 2 diabetes mellitus (S3 Table). ACR had a highly significant positive correlation with random blood sugar levels in patients with type 2 diabetes mellitus (r = 0.302; p = 0.002) and diabetic nephropathy (r = 0.323; p = 0.005). Moreover, a highly significant negative correlation was present between urinary creatinine levels and ACR in controls (r = -0.356; p = 0.001), type 2 diabetes mellitus (r = -0.331; p = 0.001), T2DM+DN (r = -0.375; p = 0.001) and T2DM-DN patients (r = -0.506; p = 0.010). On the contrary, Urinary albumin excretion was found to be directly associated with ACR levels except in T2DM-DN group (p-value >0.05) (S3 Table and Fig 1).

In the S4 and S5 Tables, according to genotypes, correlation coefficients of ACR with other parameters were determined. In the AG genotype, the urinary albumin excretion and random blood sugar levels were found to have a positive correlation in both T2DM and T2DM+DN female patients. While in male T2DM and T2DM+DN patients, UAE and ACR had a negative correlation in G genotype whereas it was positive for A genotype.

### Risk assessment of *ACE2* G8790A polymorphism with type 2 diabetes and diabetic nephropathy

For X-linked *ACE2*, male and female individuals were analyzed separately in this study (Table 2). Genotype frequencies formed by *ACE2* G8790A polymorphism in the case group were homozygous GG (8%), heterozygous AG (72%) and homozygous AA (20%) among female subjects and allelic frequencies were G (38%) and A (62%) for male subjects. A highly significant difference was observed between the genotypic and allelic frequencies of *ACE2* polymorphism between the case and control group by chi-square analysis. A significant risk association was observed between the AG (OR = 6.6 [95% CI = 2.00–21.63] p = 0.002) and AA (OR = 9.5 [95% CI = 2.075–43.502] p = 0.004) concerning GG genotype for G8790A polymorphism and the susceptibility to T2DM (p-value<0.05) in female patients. Combined analysis for genotypes revealed that AG+AA in comparison to GG increased 7–fold risk for T2DM in female subjects (p-value 0.001) which dropped to 5-fold after adjustment with multinomial logistic regression for confounding factors. Similarly, the allelic comparison revealed that A-allele was associated with increased risk of type 2 diabetes mellitus in Pakistani females (OR = 2.263 [95% CI = 1.282–3.993] p = 0.005). The risk for A-allele increased 5 times (OR = 5.132 [95% CI = 1.285–20.486] p = 0.021) when adjusted with other factors. Chi-square analysis and risk estimation through logistic regression for males in T2DM and control groups showed that allele A of *ACE2* G8790A on the X- chromosome was associated with an increased risk of T2DM (OR = 0.263 [95% CI = 0.114–0.604] p = 0.008). There was a 19-fold increment in type 2 diabetes mellitus risk in males when adjusted with confounding factors age, BMI, smoking and ACR (OR = 19.001 [95% CI = 2.641–136.68] p = 0.003). Frequency distribution analyses of all genotypes (GG/AG/AA) and alleles (A/G) at both loci between T2DM males and females with or without diabetic nephropathy showed an insignificant association which was unlikely to be a risk factor for diabetic nephropathy (Table 3). All the genotypes were found to be equally distributed in both groups, T2DM-DN and T2DM+DN for females. In contrast, a minor difference was observed in male genotypes as the frequency of the G allele was higher in T2DM+DN (43%) than in T2DM-DN (27%). The A allele frequency was higher in the T2DM-DN (73%) group than the T2DM+DN group (57%).

**Table 2:** Distribution of genotypic frequencies for G8790A polymorphism in the study population and risk analysis for type 2 diabetic mellitus.

Genotypes	Control N (%)	T2DM N (%)	χ^2^	p^a^-value	OR (CI 95%)	p^b^-value	OR^A^ (CI 95%)	p^c^-value
**Females**								
**Genotypes**	**50 (100)**	**50 (100)**						
**GG**	19 (38)	4 (8)	---		Ref (1.0)			
**AG**	26 (52)	36 (72)	11.96	0.001**	6.577 (2.00–21.63)	0.002**	5.016 (1.255–20.05)	0.023*
**AA**	5 (10)	10 (20)	9.67	0.002**	9.500 (2.075–43.502)	0.004**	5.721 (0.907–36.078)	0.063
**GG**	19 (38)	4 (8)	---		Ref (1.0)			
**AA+AG**	31 (62)	46 (92)	13.57	0.001**	7.048 (2.187–22.720)	0.001**	5.132 (1.285–20.486)	0.021*
**AG**	26 (52)	36 (72)	---		Ref (1.0)			
**AA+GG**	24 (48)	14 28()	4.28	0.038*	0.421 (0.184–0.966)	0.041*	0.459 (0.174–1.209)	0.115
**AA**	5 (10)	10 (20)	---		Ref (1.0)			
**AG+GG**	45 (90)	40 (80)	1.99	0.158	0.444 (0.140–1.411)	0.169	0.656 (0.176–2.450)	0.531
**Alleles**	**100 (100)**	**100 (100)**						
**G**	64 (64)	44 (44)	---		Ref (1.0)			
**A**	36 (36)	56 (56)	8.11	0.004**	2.263 (1.282–3.993)	0.005**	5.132 (1.285–20.486)	0.021*
**Males**								
**Alleles**	**50 (100)**	**50 (100)**						
**G (54)**	35 (70)	19 (38)	---		Ref (1.0)			
**A (46)**	15 (30)	31 (62)	10.50	0.001**	0.263 (0.114–0.604)	0.002**	19.001 (2.641–136.68)	0.003**

N = number of individuals. % Frequency is shown in parentheses. Differences in the frequencies of the genotypes between diabetic patients and controls were compared using Chi-square (*χ*^2^) test. Odds ratios with 95% confidence interval (95% CI) are presented and adjusted with other covariates. These are adjusted as OR^A^ (age, BMI, T2DM duration and Smoker/Non-smoker and ACR). p^a^-value(chi-square p-value); p^b^-value (Odd ratio p-value) and p^c^-value (Odd ratio p-value after adjustment with other covariates).

*significant at the level of 0.05 and

**highly significant at the level of 0.01.

**Table 3:** Distribution of genotypic frequencies for G8790A polymorphism in the study population and risk analysis for diabetic nephropathy.

Genotypes	T2DM-DN N (%)	T2DM+DN N (%)	χ^2^	p^a^-value	OR (95% CI)	p^b^-value
**Females**						
**Genotypes**	**11 (100)**	**39 (100)**				
**GG**	1 (9)	3 (8)	---		Ref (1.0)	
**AG**	8 (73)	28 (72)	0.016	0.900	1.167 (0.106–12.805)	0.900
**AA**	2 (18)	8 (20)	0.042	0.839	1.333 (0.086–20.707)	0.837
**GG**	1 (9)	3 (8)	---		Ref (1.0)	
**AA+AG**	10 (91)	36 (92)	0.022	0.882	1.20 (0.112–12.826)	0.880
**AG**	8 (73)	28 (72)	---		Ref (1.0)	
**AA+GG**	3 (27)	11 (28)	0.004	0.951	1.048 (0.234–4.691)	0.951
**AA**	2 (18)	8 (20)	---		Ref (1.0)	
**AG+GG**	9 (82)	31 (80)	0.030	0.863	0.861 (0.154–4.799)	0.865
**Alleles**	**22 (100)**	**78 (100)**				
**G**	10 (45)	34 (44)	---		Ref (1.0)	
**A**	12 (55)	44 (56)	0.024	0.876	1.078 (0.417–2.792)	0.876
**Males**						
**Alleles**	**15 (100)**	**35 (100)**				
**G (54)**	4 (27)	15 (43)	---		Ref (1.0)	
**A (46)**	11 (73)	20 (57)	0.753	0.386	1.786 (0.470–6.789)	0.395

N = number of individuals. % Frequency is shown in parentheses. T2DM+DN: Type 2 diabetes with diabetic nephropathy, T2DM-DN: Type 2 diabetes without diabetic nephropathy. Differences in the frequencies of the genotypes between diabetic patients with or without diabetic nephropathy were compared using Chi-square (*X*^2^) test and without adjusting for other covariates. Odds ratios with 95% confidence interval (95% CI) are presented and adjusted with other covariates. p^a^_-_value (chi-square p-value); p^b^-value (Odd ratio p-value).

*significant at the level of 0.05 and

**highly significant at the level of 0.01.

### Association of *ACE2* G8790A polymorphisms with clinical parameters

**Fig 2:**
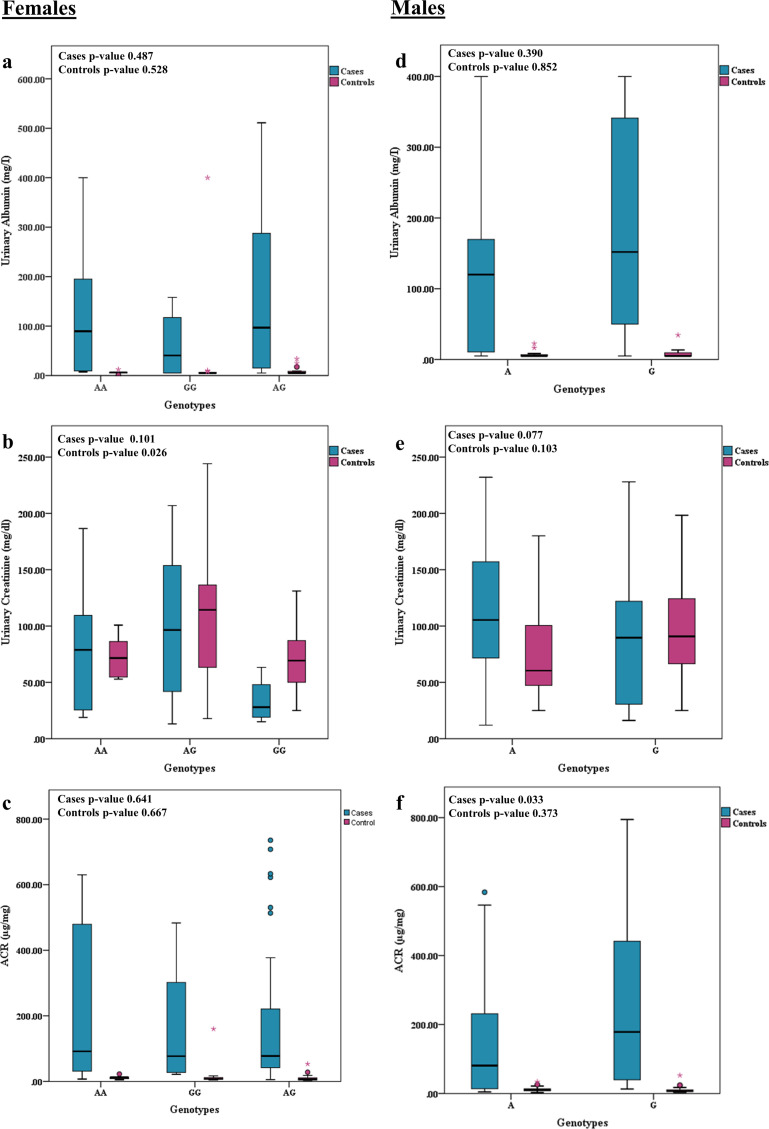
Association of G8790A *ACE2* polymorphisms with the distribution of UAE, Urinary Creatinine and ACR data in controls and T2DM cases. **a-c)** Distribution of data according to female genotypes (GG/AG/AA) **d-f)** Distribution of data according to male genotypes (A/G).

By the influence of *ACE2* G8790A polymorphism genotypes on age, BMI, blood pressure and clinical biochemical variables among type 2 diabetes mellitus patients, it was explored that all these factors were not related to the genotypes in the T2DM females except urinary creatinine which showed a significant difference in the recessive model [GGⅹ (AG+AA); p-value 0.049] (Table 4). In males, the duration of diabetes showed significant distribution between G and A alleles while no significant association was found for all other variables (Table 5). In Fig 2, the differences between urinary albumin excretions, urinary creatinine and ACR data distribution according to genotypes in case and control groups had been presented. Only urinary creatinine levels were found different in control group genotypes (p-value 0.026) where AG genotype had higher urinary creatinine levels in comparison to the other two genotypes (GG and AA) (Fig 2B). Among all the other comparisons, data was evenly distributed in genotypes of both normal and diabetic males and females (Fig 2).

**Table 4:** Analysis of the influence of *ACE2* G8790A polymorphism on clinical-biochemical variables in female T2DM patients.

Characteristics	T2DM				p-value	p-value
	GG	AG	AA	AG+AA	GGⅹAGⅹAA	GGⅹ[AG+AA]
**Age (years)**	51.75±2.99	53.06±7.40	53.90±5.59	53.24±6.99	0.856	0.677
**BMI (Kg/m** ^**2**^ **)**	26.75±3.59	30.59±6.66	31.05±2.95	30.69±6.02	0.443	0.206
**T2DM duration (<10) years)**	16.75±4.99	13.61±3.51	15.5±3.57	14.02±3.57	0.134	0.161
**SBP (mmHg)**	130.0±14.14	124.72±13.63	130.0±13.33	125.87±13.59	0.474	0.564
**DBP (mmHg)**	82.50±5.00	78.9±8.87	82.0±9.18	79.57±8.93	0.50	0.523
**Pulse Rate (per minute)**	75.00±5.03	78.78±6.16	77.70±6.62	78.50±6.21	0.476	0.280
**Random Blood Sugar (mg/dl)**	228.00±84.70	238.00±104.84	231.50±85.98	236.59±100.17	0.971	0.869
**Urinary creatinine (mg/dl)**	33.55±21.12	98.78±61.08	80.84±55.52	94.89±59.79	0.101	0.049*
**UAE (mg/l)**	61.07±72.83	150.99±152.01	162.39±155.25	153.47±151.05	0.487	0.235
**ACR (mg/g)**	164.65±216.90	183.13±219.18	255.55±248.23	198.88±224.95	0.641	0.771

Analysis by ANOVA (GGxAGxAA) and Student t-test (GGx[AG+AA]).

*significant at the level of 0.05 and **highly significant at the level of 0.01.

**Table 5:** Analysis of the influence of *ACE2* G8790A polymorphism on clinical -biochemical variables in male T2DM patients.

Characteristics:	T2DM		p-value
	G	A	
**Age (years)**	58.32±7.11	56.90±7.69	0.520
**BMI (Kg/m** ^**2**^ **)**	26.48±4.51	27.39±7.13	0.621
**T2DM duration (<10) years)**	15.11±5.26	14.87±5.89	0.888
**SBP (mmHg)**	119.79±16.01	121.9±14.01	0.621
**DBP (mmHg)**	77.37±6.53	79.03±9.44	0.503
**Pulse Rate (per minute)**	79.68±5.97	79.03±7.06	0.739
**Random Blood Sugar (mg/dl)**	252.00±111.23	231.13±64.13	0.403
**Urinary creatinine (mg/dl)**	96.83±68.90	112.50±57.54	0.077
**UAE (mg/l)**	182.32±155.71	110.66±123.19	0.390
**ACR (mg/g)**	254.57±248.33	128.61±158.93	0.033**

Analysis by independent sample t-test.

*significant at the level of 0.05 and

**highly significant at the level of 0.01.

**Fig 3:**
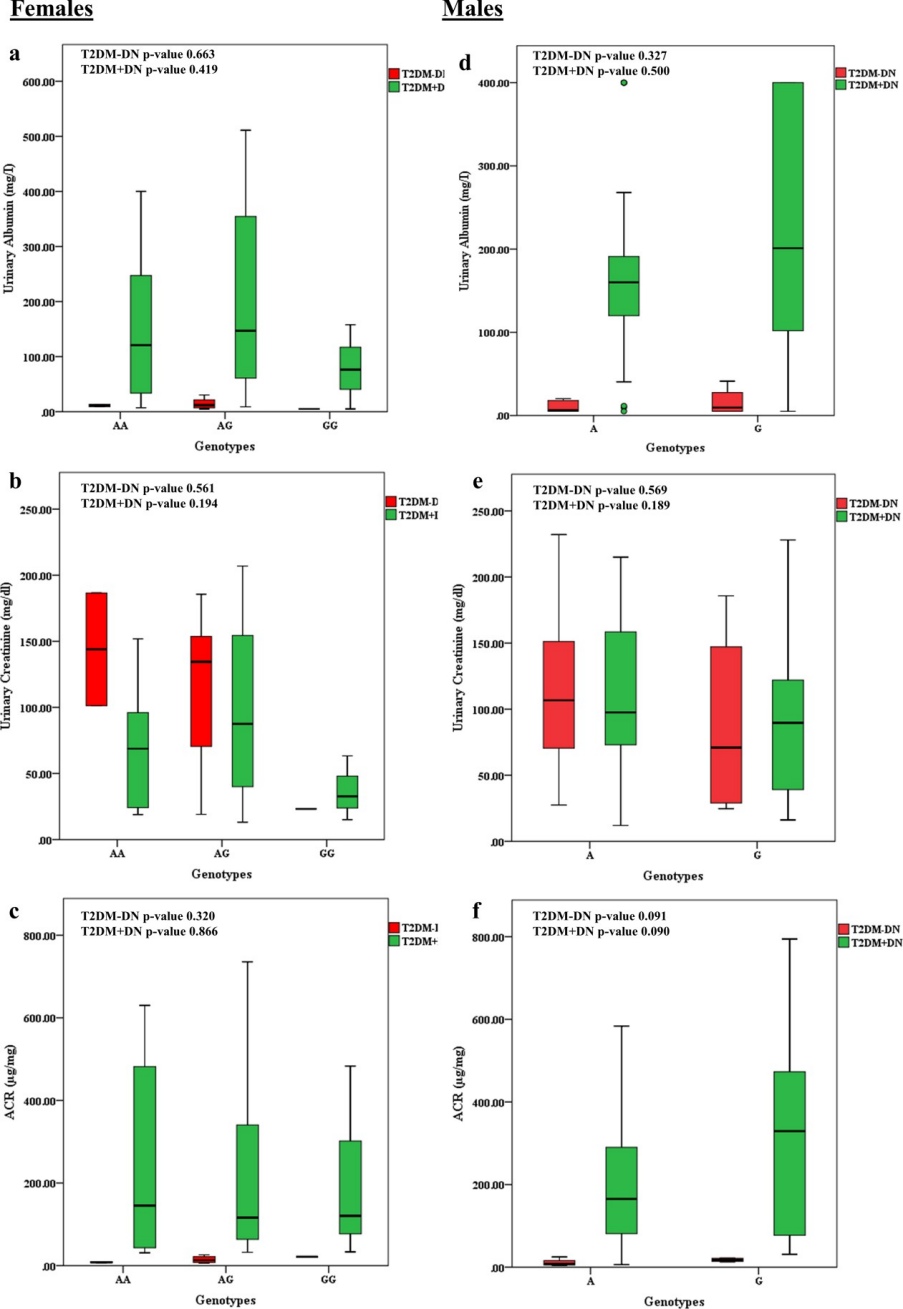
Association of G8790A *ACE2* polymorphisms with the distribution of UAE, Urinary Creatinine and ACR data in T2DM patients with or without diabetic nephropathy. **a-c)** Distribution of data according to female genotypes (GG/AG/AA) **d-f)** Distribution of data according to male genotypes (A/G).

Among diabetic nephropathic female patients, there was a significant difference found in the diabetes duration between all three genotypes GG, AG and AA (p-value 0.05). The highest mean duration was associated with the GG genotype (18.00±5.29) while the lowest one was associated with the AG genotype (52.36±6.87) followed by the AA genotype (52.25±4.95). While all the other variables had an insignificant distribution in association with genotypes among T2DM+DN female and male patients (Tables 6 and 7) (Fig 3).

**Table 6:** Analysis of the influence of ACE2 G8790A polymorphism on clinical-biochemical variables in female T2DM patients with diabetic nephropathy.

Characteristics	T2DM+DN				p-value	p-value
	GG	AG	AA	AG+AA	GGⅹAGⅹAA	GGⅹ[AG+AA]
**Age (years)**	50.67±2.52	52.36±6.87	52.25±4.95	52.33±6.428	0.909	0.661
**BMI (Kg/m** ^**2**^ **)**	25.13±1.90	30.57±6.25	31.28±2.85	30.73±5.65	0.250	0.09
**T2DM duration (<10 years)**	18.00±5.29	13.57±3.12	15.9±3.79	14.08±3.37	0.051*	0.07
**SBP (mmHg)**	130.0±17.32	124.29±14.5	130.0±15.12	125.56±14.63	0.560	0.620
**DBP (mmHg)**	83.33±5.77	79.64±9.62	81.25±9.91	80.00±9.56	0.773	0.559
**Pulse Rate (per minute)**	77.33±2.31	79.43±6.30	77.50±7.39	79.00±7.49	0.687	0.664
**Random Blood Sugar (mg/dl)**	208.33±91.87	242.32±113.90	256.50±75.16	245.47±105.71	0.800	0.560
**Urinary creatinine (mg/dl)**	37.02±24.44	94.09±61.70	68.59±46.77	88.42±59.07	0.194	0.147
**UAE (mg/l)**	79.77±76.56	189.98±151.12	151.34±140.02	181.39±147.66	0.419	0.250
**ACR (mg/g)**	212.33±238.59	231.29±226.80	250.28±241.46	235.51±226.73	0.966	0.866

Analysis by one way ANOVA (GGxAGxAA) and Student t-test (GGx [AG+AA]).

*significant at the level of 0.05 and

**highly significant at the level of 0.01.

**Table 7:** Analysis of the influence of ACE2 G8790A polymorphism on clinical-biochemical variables in male T2DM patients with diabetic nephropathy.

Characteristics:	T2DM+DN		p-value
	G	A	
**Age (years)**	58.00±8.02	56.45±7.67	0.566
**BMI (Kg/m** ^**2**^ **)**	26.95±4.81	27.89±8.69	0.706
**T2DM duration (<10 years)**	15.47±5.40	14.95±6.83	0.811
**SBP (mmHg)**	123.73±15.14	125.00±12.77	0.790
**DBP (mmHg)**	78.67±5.16	80.50±6.86	0.393
**Pulse Rate (per minute)**	79.60±5.67	81.80±6.15	0.287
**Random Blood Sugar (mg/dl)**	262.53±116.09	228.95±65.40	0.285
**Urinary creatinine (mg/dl)**	99.17±69.74	113.98±58.61	0.189
**UAE (mg/l)**	226.58±145.39	165.98±121.92	0.500
**ACR (mg/g)**	317.76±242.82	198.71±160.58	0.090

Analysis by independent sample t-test. *significant at the level of 0.05 and **highly significant at the level of 0.01.

## Discussion

Diabetic nephropathy (DN) is the long time existing complication of type 2 diabetes mellitus. In this study, 100 healthy controls and 100 patients with type 2 diabetes mellitus (T2DM) were enrolled and their data were analyzed. The T2DM 100 patients were further categorized into two groups which had chronic diabetes with or without diabetic nephropathy. The present study investigated the genetic association of *ACE2* G8790A polymorphism with the risk, clinical findings and progression of the disease. The results indicated that *ACE2* G8790A polymorphism had considerable association with type 2 diabetes mellitus but not with diabetic nephropathy in combination with T2DM. Several past pieces of research investigated the association of this polymorphism with either T2DM or other diabetic complications such as cerebral stroke, retinopathy, cardiovascular disease, and hypertension. In 2006, a study conducted on T2DM patients of Chinese Han and Dongxiang ethnicity reported that in *ACE2* G8790A polymorphism, A allele (p-value = 0.049) and AA genotype (p-value = 0.001) were more prevalent among T2DM subjects who also had essential hypertension [22]. The *ACE2* AA genotype and A allele were significantly associated with the risk of cerebral stroke in type 2 diabetes mellitus patients [OR = 3.733 (2.069–6.738), OR = 3.597 (1.884–6.867)] [23]. Contrasting findings were observed in another study where greater percentages of GG genotype and G allele were associated with CAD (coronary artery disease) in diabetic patients of Caucasian origin and *ACE2* genotyping was proven to be helpful in screening of the susceptible patients [24]. The results of a research study on the Han Chinese population indicated that common genetic variants of the *ACE2* gene including G8790A might impact myocardial infarction in females, and may interact with alcohol consumption to increase the risk of chronic heart disease and myocardial infarction in male subjects [25]. A recent research study in the Brazilian population proved that a combination of *ACE* insertion/deletion (I/D) and *ACE2* G8790A polymorphisms are an effective genetic marker for systemic arterial hypertension (SAH) in type 2 diabetes mellitus patients with the susceptibility profile of DD/GG genotype in females of studied groups. The DD genotype represents a deletion of an Alu sequence in intron 16 of the ACE gene while the GG genotype signifies the G8790A single nucleotide polymorphism of the *ACE2* gene [26]. In another study by *Gintoni et*. *al*, the G8790A (rs228566) functional polymorphism of *ACE2* association with basal cell carcinoma (BCC) had been studied. A total of 190 DNA samples (91 patients and 99 controls) of Greek origin had been studied, yet they found no association between *ACE2* G8790A polymorphism and BCC pathogenesis [27]. The findings from this study were found in line with the present study as no significant association was observed between *ACE2* G8790A polymorphism and potential risk of diabetic nephropathy. Irbesartan is a drug for the treatment of hypertension (stage II) associated with chronic renal failure. In a Chinese study, the effect of G8790A polymorphism on the Irbesartan treatment was investigated. A decline was observed in blood pressure for the TT genotype following Irbesartan treatment compared with TC and CC genotype (p-value 0.018). However, no difference was found between renal function levels in pre and post-treatment results. In line with the present study, no association was found between G8790A polymorphisms and renal function improvement following Irbesartan treatment although it may influence the anti-hypertensive effect of the drug [28].

Our results also indicate a negative association between *ACE2* G8790A and the development of T2DM with or without DN in comparison to other confounding factors. A high percentage of AG genotype in females and A allele in males are associated with T2DM in the general population of Pakistan. Recently, a group of scientists from Spain had investigated the impact of *ACE2* G8790A polymorphism on the outcome of COVID-19 in a short communication. Their work concluded that there was no association between *ACE2* polymorphism and disease outcome because sequencing results presented no coding sequence variants which could be suggestive of developing disease risk [29].

*Firouzabadi et al*. had investigated the role of *ACE2* G8790A variant against selective serotonin reuptake inhibitors (SSRIs) in a randomized control trial of the Iranian population who had been newly diagnosed with major depressive disorder and completed 6 weeks of treatment with sertraline or fluoxetine. They found that AG and AA genotypes [OR = 3.3 (1.2–1.9) p-value = 0.008] and the A allele [OR = 3.4 (1.4–8.5) p-value = 0.027] were remarkably associated with better response to drugs. On the contrary to the present study, they concluded that the G8790A polymorphism had a role in response to some SSRIs [30].

We had also correlated ACR with age, BMI and clinical parameters to investigate their influence on the occurrence of type 2 diabetes mellitus and diabetic nephropathy. In T2DM and T2DM+DN patients when random blood sugar levels and UAE increased, ACR values had also been elevated. Whereas, urinary creatinine levels were negatively correlated with ACR which is suggestive of the observation that increase in Urinary creatinine levels caused a decline in ACR. Although these results were conflicting with Idowu et al. findings because they found a positive correlation of urinary creatinine and ACR in microalbuminuric diabetic patients who had >5–10 years duration of disease. According to that study, ACR levels were elevated with increase in duration of diabetes which leads to progression of microalbuminuria towards macroalbuminuria [31]. The strength of the present study lies in the utilization of genotyping method that was simple, economical and robust. Furthermore, the studied population sample was representative of Pakistani Type 2 diabetic patients with or without diabetic nephropathy. It had been previously demonstrated that ACE2 expression is altered in human and rat kidneys with diabetes [32, 33] thus it had hypothesized that gene variants could be associated with diabetes and diabetic nephropathy. ACE2 is an enzyme with potential catalytic efficiency that cleaves Ang-II to form Ang 1–7 peptides, thus inhibiting profibrotic and adverse vasoconstrictive effects of AngII. The formation of Ang 1–7 minimizes the oxidative stress on endothelial cerebral arteries and ultimately acts as a vasodilator and antifibrotic [34]. The presence of mutations in the *ACE2* gene in the form of polymorphisms cause the absence of ACE2 enzyme which results in a reduced level of Ang 1–7 in serum and consequently, the protective effect on kidneys is reduced that leads towards the development of diabetic nephropathy [35]. However, any such association could not be detected by this study. The possibility behind this non-association may be that location of this G8790A SNP is an intronic region of the *ACE2* gene which may not alter the *ACE2* gene expression to form a fully functional ACE2 enzyme and eventually level of Ang 1–7 in serum remain constant. Alternatively, a linkage could be present between genetic polymorphisms in the transcription unit and regulatory region of *ACE2* gene and ACE2 enzyme which affects susceptibility of type 2 diabetes mellitus and diabetic nephropathy. Further studies using a large number of patients and correlating the polymorphism with serum ACE2 levels are required to further clarify the role of *ACE2* polymorphism in type 2 diabetes and diabetic nephropathy.

## Conclusion

According to the results obtained in this study, it can be concluded that AG and AA genotypes of *ACE2* (G8790A) polymorphism had a statistically significant risk associated with type 2 diabetes. The final findings of the study showed a non-significant association of *ACE2* polymorphism with diabetic nephropathy in the Pakistani population. Hence, there is a need to analyze the association of ACE2 with diabetic nephropathy either in combination with other genes and polymorphisms or on a large scale of data. Moreover, it was found that ACR had a positive correlation with urinary albumin and random blood sugar levels through which we hypothesized that by managing random blood sugar levels, albumin to creatinine ratio can also be maintained which is directly associated with microalbuminuria that further leads to diabetic nephropathy.

## Supporting information

S1 Table Demographic, clinical and genotypic data of 100 patients with type 2 diabetes and 100 controls without type 2 diabetes for this study. (XLSX)

S2 Table Distribution of demographic features, anthropometric measurements and clinical parameters by gender in the present study. (PDF)

S3 Table The correlation coefficient analysis of ACR with other parameters among study population groups. (PDF)

S4 Table The correlation coefficient analysis of ACR with other parameters in type 2 diabetes mellitus male and female patients according to genotypes. (PDF)

S5 Table The correlation coefficient analysis of ACR with other parameters in diabetic nephropathy male and female patients according to genotypes. (PDF)
