# Supplementary material for: Investigation of angiotensin-1 converting enzyme 2 gene (G8790A) polymorphism in patients of type 2 diabetes mellitus with diabetic nephropathy in Pakistani population
Source: PLoS One. 2022 Feb 17;17(2):e0264038. doi: 10.1371/journal.pone.0264038 (PMC8853542; doi:10.1371/journal.pone.0264038)
Supplement: S2 Table — (PDF) [file pone.0264038.s002.pdf]

Table S1: Distribution of demographic features, anthropometric measurements and clinical parameters by gender in the present study.

	**Controls**			**Cases**		
**Characteristics:**	**Male (N=50)**	**Female (N=50)**	**p-value**	**Male (N=50)**	**Female (N=50)**	**p-value**
**Age (years)**	50.88±6.94	49.56±5.8	0.305	57.84±7.34	52.88±7.15	0.001**
**BMI (Kg/m^2)**	27.89±3.74	28.06±3.22	0.817	28.84±6.40	29.83±5.52	0.407
**Smoking Status**						
**Smokers**	0 (0 %)	0 (0 %)		9 (9 %)	0 (0 %)	0.002**
**Non-Smokers**	50 (91 %)	50 (100 %)		41 (91 %)	50 (100 %)	
**T2DM duration (<10 years)**	----	----	----	15.08±5.56	14.12±3.75	0.314
**SBP (mmHg)**	120.00±0.00	120.00±0.00	----	121.52±14.64	125.80±13.72	0.135
**DBP (mmHg)**	80.00±0.00	80.00±0.00	----	78.40±8.42	79.80±8.69	0.415
**Pulse Rate (per minute)**	79.84±5.74	78.58±6.92	0.325	79.28±6.61	78.22±6.16	0.409
**Random Blood Sugar (mg/dl)**	116.96±12.85	113.10±16.41	0.193	238.02±85.28	236.94±97.77	0.953
**Urinary creatinine (mg/dl)**	90.48±43.83	90.39±44.90	0.978	105.98±62.07	90.54±59.91	0.209
**UAE (mg/l)**	7.77±5.29	7.74±7.05	0.992	145.70±142.95	138.26±144.65	0.796
**ACR (mg/g)**	10.68±8.94	12.72±22.80	0.556	189.29±208.46	185.40±218.02	0.928

Data are shown as Mean ± SD. BMI; body mass index, SBP; systolic blood pressure, DBP; diastolic blood pressure, UAE; urinary albumin excretion, ACR; Albumin to creatinine ratio, T2DM; type 2 diabetes mellitus. The p-value: *significant at the level of 0.05 and **significant at the level of 0.01.
