# Supplementary material for: Investigation of angiotensin-1 converting enzyme 2 gene (G8790A) polymorphism in patients of type 2 diabetes mellitus with diabetic nephropathy in Pakistani population
Source: PLoS One. 2022 Feb 17;17(2):e0264038. doi: 10.1371/journal.pone.0264038 (PMC8853542; doi:10.1371/journal.pone.0264038)
Supplement: S3 Table — (PDF) [file pone.0264038.s003.pdf]

Table S3: The correlation coefficient analysis of ACR with other parameters among study population groups.

Parameters	Controls r-value (p-value)	T2DM r-value (p-value)	T2DM-DN r-value (p-value)	T2DM+DN r-value (p-value)
**Age (years)**	-0.088 (0.385)	-0.061 (0.549)	0.041 (0.845)	0.032 (0.786)
**T2DM Duration (<years)**	-------	-0.001 (0.993)	0.258 (0.213)	-0.042 (0.722)
**BMI (Kg/m^2)**	-0.159 (0.114)	-0.024 (0.816)	-0.012 (0.954)	-0.075 (0.522)
**SBP (mmHg)**	-------	0.182 (0.070)	0.086 (0.628)	0.117 (0.316)
**DBP (mmHg)**	-------	0.212 (0.034)	-0.101 (0.630)	0.179 (0.125)
**Pulse Rate (per minute)**	-0.030 (0.770)	0.158 (0.116)	-0.329 (0.108)	0.031 (0.792)
**Random Blood Sugar (mg/dl)**	0.049 (0.628)	0.302 (0.002)**	0.100 (0.635)	0.323 (0.005)**
**Urinary creatinine (mg/dl)**	-0.356 (0.001)**	-0.331 (0.001)**	-0.506 (0.010)**	-0.375 (0.001)**
**UAE (mg/l)**	0.655 (0.001)**	0.642 (0.001)**	0.353 (0.083)	0.524 (0.001)**

BMI; body mass index, SBP; systolic blood pressure, DBP; diastolic blood pressure, UAE; urinary albumin excretion, ACR; Albumin to creatinine ratio, T2DM; type 2 diabetes mellitus. The r-value is Pearson correlation and the p-value is *significant at the level of 0.05 and **highly significant at the level of 0.01.
