# Supplementary material for: Investigation of angiotensin-1 converting enzyme 2 gene (G8790A) polymorphism in patients of type 2 diabetes mellitus with diabetic nephropathy in Pakistani population
Source: PLoS One. 2022 Feb 17;17(2):e0264038. doi: 10.1371/journal.pone.0264038 (PMC8853542; doi:10.1371/journal.pone.0264038)
Supplement: S4 Table — (PDF) [file pone.0264038.s004.pdf]

Table S4: The correlation coefficient analysis of ACR with other parameters in type 2 diabetes mellitus male and female patients according to genotypes.

Parameters	Females Genotypes			Males Genotypes	
	GG (n=4) r-value (p-value)	AG (n=36) r-value (p-value)	AA (n=10) r-value (p-value)	G (n= 19) r-value (p-value)	A (n=31) r-value (p-value)
**Age (years)**	-0.072 (0.928)	-0.118 (0.494)	-0.365 (0.300)	-0.083 (0.736)	0.092 (0.621)
**Duration of T2DM**	0.605 (0.395)	0.087 (0.612)	0.363 (0.302)	-0.127 (0.606)	-0.152 (0.413)
**BMI (Kg/m^2)**	-0.538 (0.462)	-0.154 (0.369)	0.241 (0.503)	0.133 (0.589)	0.054 (0.774)
**SBP (mmHg)**	-0.299 (0.701)	0.075 (0.665)	0.434 (0.210)	0.271 (0.261)	0.284 (0.122)
**DBP (mmHg)**	-0.136 (0.864)	0.219 (0.200)	0.442 (0.201)	0.284 (0.239)	0.176 (0.343)
**Pulse Rate (per minute)**	0.189 (0.811)	0.022 (0.898)	0.069 (0.851)	0.136 (0.579)	0.484 (0.006)**
**Random Blood Sugar (mg/dl)**	-0.838 (0.162)	0.472 (0.004)**	-0.195 (0.589)	0.362 (0.128)	0.072 (0.700)
**UAE (mg/l)**	0.962 (0.038)*	0.560 (0.001)**	0.729 (0.017)*	-0.585 (0.009)**	0.785 (0.001)**
**Urinary creatinine (mg/dl)**	0.170 (0.830)	-0.350 (0.036)*	-0.384 (0.273)	-0.396 (0.093)	-0.276 (0.132)

BMI; body mass index, SBP; systolic blood pressure, DBP; diastolic blood pressure, UAE; urinary albumin excretion, ACR; Albumin to creatinine ratio, T2DM; type 2 diabetes mellitus. The r-value is Pearson correlation and the p-value is *significant at the level of 0.05 and **highly significant at the level of 0.01.
