# Supplementary material for: Investigation of angiotensin-1 converting enzyme 2 gene (G8790A) polymorphism in patients of type 2 diabetes mellitus with diabetic nephropathy in Pakistani population
Source: PLoS One. 2022 Feb 17;17(2):e0264038. doi: 10.1371/journal.pone.0264038 (PMC8853542; doi:10.1371/journal.pone.0264038)
Supplement: S5 Table — (PDF) [file pone.0264038.s005.pdf]

Table S5: The correlation coefficient of ACR with other parameters in diabetic nephropathy male and female patients according to genotypes.

Parameters	Females Genotype			Males Genotype	
	GG (n=3)	AG (n=28)	AA (n=8)	G (n=15)	A (n=21)
**Age (years)**	0.294 (0.810)	-0.065 (0.743)	-0.404 (0.321)	-0.036 (0.899)	0.229 (0.187)
**Duration of T2DM**	0.494 (0.671)	0.129 (0.514)	0.182 (0.666)	-0.254 (0.361)	-0.186 (0.419)
**BMI (Kg/m^2)**	-0.345 (0.776)	-0.235 (0.229)	0.452 (0.260)	0.113 (0.690)	-0.088 (0.706)
**SBP (mmHg)**	-0.333 (0.784)	0.115 (0.561)	0.382 (0.350)	0.033 (0.906)	0.204 (0.375)
**DBP (mmHg)**	-0.333 (0.784)	0.176 (0.371)	0.630 (0.114)	0.140 (0.619)	0.103 (0.656)
**Pulse Rate (per minute)**	-0.650 (0.550)	-0.072 (0.715)	-0.221 (0.600)	0.210 (0.452)	0.305 (0.179)
**Random Blood Sugar (mg/dl)**	-0.796 (0.414)	0.560 (0.006)**	-0.208 (0.622)	0.337 (0.219)	0.173 (0.452)
**UAE (mg/l)**	0.955 (0.191)	0.450 (0.016)*	0.667 (0.071)	0.420 (0.119)	0.677 (0.001)**
**Urinary creatinine (mg/dl)**	0.030 (0.981)	-0.352 (0.066)	-0.206 (0.625)	-0.560 (0.030)*	-0.413 (0.063)

BMI; body mass index, SBP; systolic blood pressure, DBP; diastolic blood pressure, UAE; urinary albumin excretion, ACR; Albumin to creatinine ratio, T2DM; type 2 diabetes mellitus. The r-value is Pearson correlation and the p-value is *significant at the level of 0.05 and **highly significant at the level of 0.01.
